# Intraplacental choriocarcinoma coexisting with fetomaternal hemorrhage and fetal intrahepatic portosystemic venous shunt in a term pregnancy: A case report

**DOI:** 10.1097/MD.0000000000044208

**Published:** 2025-08-29

**Authors:** Yi Sun, Pei Zhou, Yongpan Tan, Tao Liu, Xingxing Wang, Lufang Wang

**Affiliations:** a Department of Ultrasound, The Fourth Hospital of Shijiazhuang, Shijiazhuang, Hebei, China.

**Keywords:** fetomaternal hemorrhage, intrahepatic portosystemic venous shunt, intraplacental choriocarcinoma, middle cerebral artery peak systolic velocity, ultrasound

## Abstract

**Rationale:**

Intraplacental choriocarcinoma (IC) coexisting with fetomaternal hemorrhage (FMH) in term pregnancy is rare and life-threatening for the mother and baby. The limited knowledge of this disease leads to delayed or missed diagnosis. This case report aims to illustrate how to avoid missed diagnosis through a more complicated case by the presence of fetal intrahepatic portosystemic venous shunt (IPSVS).

**Patient concerns:**

A 39-week pregnant woman presented to our hospital with decreased fetal movements.

**Diagnoses:**

Prenatally, the ultrasound examination identified the presence of IPSVS. FMH was diagnosed immediately after delivery, and the diagnosis was subsequently revised to IC with FMH 41 days postpartum when the mother developed symptoms of vaginal bleeding.

**Interventions:**

The mother received chemotherapy. Surveillance was conducted through measurements of beta human chorionic gonadotropin (β-hCG) and computed tomography imaging. The infant underwent β-hCG testing and ultrasound examinations for IPSVS.

**Outcomes:**

Following 6 cycles of chemotherapy, the mother’s β-hCG levels normalized, with complete resolution of pulmonary metastases. The infant’s β-hCG test results were negative, and no significant change was observed in the IPSVS.

**Lessons:**

IC coexisting with FMH is rare. Enhancing the understanding of the manifestations of this disease is vital for its early diagnosis. When unexplained FMH occurs, a systematic investigation into potential etiologies is necessary, and clinicians should remain vigilant for the possibility of IC. This case underscores the importance of thorough placental pathological examination and postpartum HCG monitoring for patients with FMH.

## 1. Introduction

Intraplacental choriocarcinoma (IC) is a focal gestational choriocarcinoma that is confined to the placenta, but with highly aggressive characteristic. IC accounts for no more than 0.04% of all gestational trophoblastic disease (GTD), and its coexistence with a term pregnancy is rare.^[1]^ In addition, IC has an occult onset and is typified by small lesions and an infarct- or thrombus-like appearance, it is easy to be overlooked, even in placental examination.^[2]^ Based on the above facts, its early diagnosis is still a challenge in clinic. Fetomaternal hemorrhage (FMH), which occurs in 38% of patients with IC, is an important clue to the diagnosis of IC.^[3]^ However, clinicians have limited knowledge of it and fail to conduct targeted examinations, resulting in missed diagnosis.

In this report, we present a case of delayed diagnosis of IC coexisting with FMH. Different from previously reported cases, in this case, the fetus also suffered from intrahepatic portosystemic venous shunt (IPSVS) which affected blood circulation, further complicating the condition.

## 2. Case presentation

**Figure 1.**
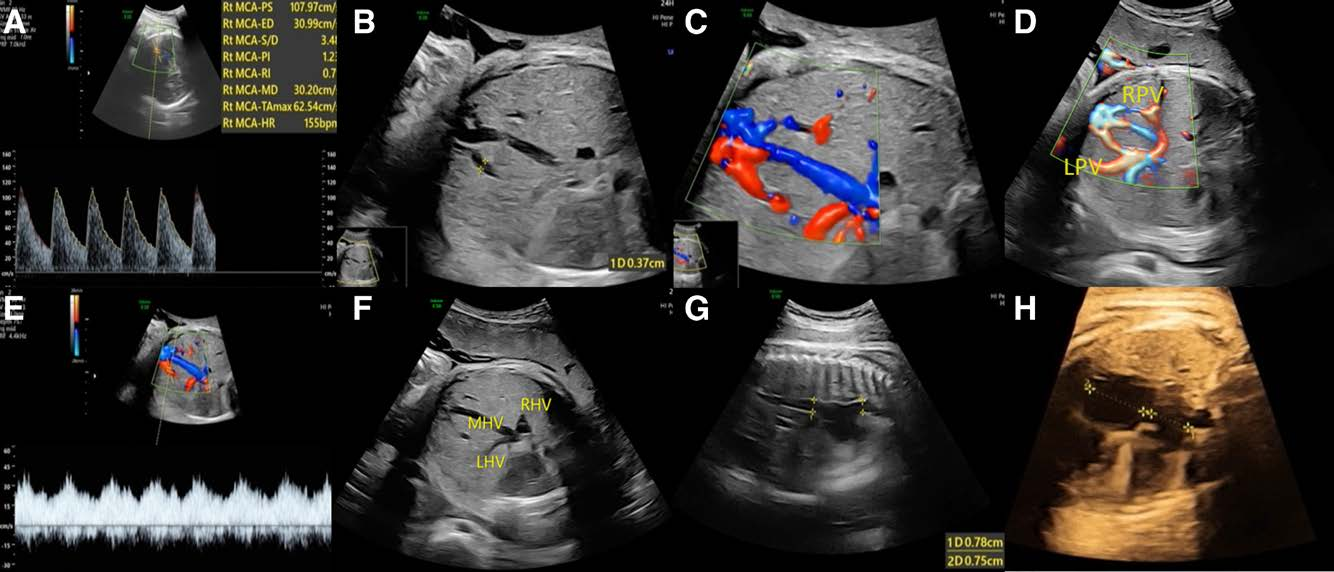
Images of measurement of MCA-PSV and IPSVS (A) markedly increased MCA-PSV. (B–E) abnormal channels between both LPV and RPV and MHV with the maximum width of 0.37 cm. (E) The collateral vessel showed typical triphasic waveform of MPV. (F, G) Expanded HV, IVC and SVC. (H) Enlarged RA. HV = hepatic vein, IPSVS = intrahepatic portosystemic venous shunt; IVC = inferior vena cava; LPV = left portal vein; MHV = middle hepatic vein, MCA-PSV = middle cerebral artery peak systolic velocity, MPV = main portal vein, RA = right atrium, RPV = right portal vein, SVC = superior vena cava.

A 31-year-old pregnant woman, gravida 1 para 0, experienced an uneventful pregnancy until 28 weeks of gestation, except for a mild COVID-19 infection at 3 months. Following this, the patient did not adhere to medical recommendations for routine prenatal examinations. At 38 weeks and 5 days of gestation, she presented to the hospital with complaints of decreased fetal movements. Cardiotocographic monitoring of the fetus showed a sinusoidal pattern. An emergency ultrasound examination was performed immediately and revealed an increase in middle cerebral artery peak systolic velocity (MCA-PSV) with a value of 107 cm/s, exceeding 1.55 multiples of the median. Additionally, abnormal vascular connections were identified between the left and right branches of the portal vein and the middle hepatic vein, with a maximum diameter of 0.37 cm; this finding was diagnosed as IPSVS. The ultrasound also revealed dilation of the hepatic vein, inferior vena cava, and superior vena cava, as well as right atrial enlargement, all of which were attributed to the hemodynamic effects of IPSVS (Fig. 1).

Due to fetal distress, emergency cesarean section was performed after ultrasound examination and a male infant weighing 3350 g was delivered. He had Apgar scores of 8, 8, and 8 at 1, 5, and 10 minutes after delivery. The infant was pale, had dyspnea, and was transferred to the neonatal department. His initial hemoglobin concentration was 46 g/L, indicating the need for an immediate blood transfusion. FMH was suspected after other causes were excluded, and was confirmed by flow cytometric analysis of maternal blood, which detected 3.02% fetal red blood cells. The infant’s IPSVS remained stable compared with prenatal assessment. Due to the small shunt flow, regular ultrasound examination is needed for follow-up, and most cases can have abnormal shunt vascular closure. After 13 days of neonatal care, the infant had normal hemoglobin levels and was discharged.

Considering the occurrence of unexplained FMH, the placenta was sent for histopathological examination. The macroscopic examination of the placenta was normal. Microscopically, there were focal thrombosis under the chorion, increased perivillous fibrin deposition and villous nodules. Besides, low-grade chronic chorioamnionitis and umbilical phlebitis were also identified.

**Figure 2.**
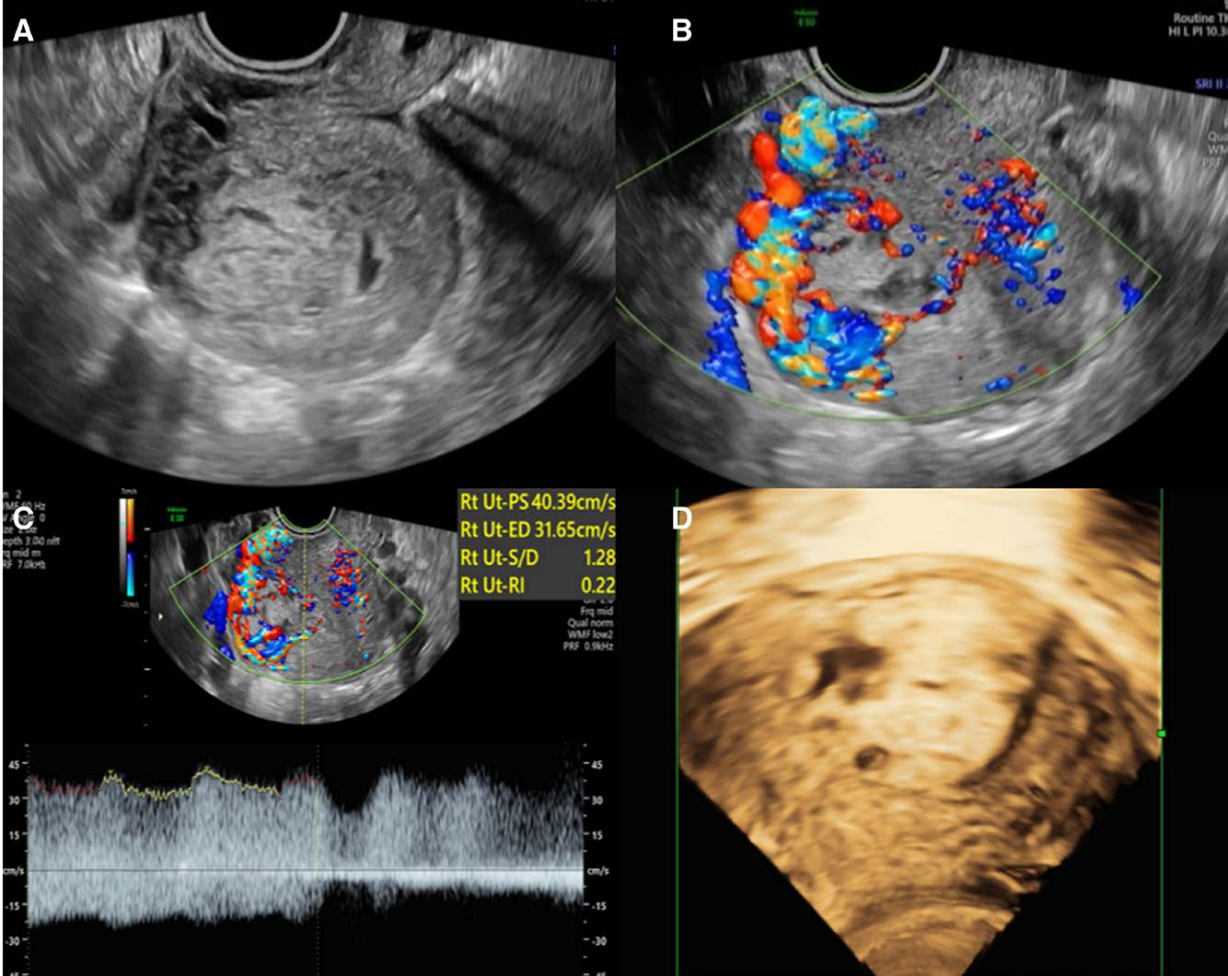
Transvaginal images of choriocarcinoma. (A) A mass in uterine cavity with heterogeneous echo texture and its boundary with normal uterine wall was not clear. (B, C) Extremely rich blood flow signal inside and surround the lesion with a low RI of 0.22. (D) 3D volume imaging of the lesion. 3D = 3-dimensional, RI = resistance index.

The mother stabilized and was discharged home 5 days after surgery. At 41 days postpartum, she presented to the hospital with vaginal bleeding. Transvaginal ultrasound examination of her uterus showed a mass measuring 3.3 cm × 2.2 cm on the right side of the uterine cavity with a heterogeneous echotexture. The boundary of this mass with the normal uterine wall was not clear (Fig. 2), and it had a rich blood supply with a low resistance index of 0.22. The patient’s serum beta human chorionic gonadotropin (β-hCG) level was elevated (>2,66,400 IU/L). Further examination revealed multiple nodules (all ≤3 mm in diameter) and ground glass density shadows in both lungs, indicating metastases. After multidisciplinary consultation, the patient was diagnosed with choriocarcinoma (FIGO score: 7). The presence of FMH without obvious inducement suggested that the choriocarcinoma had developed during pregnancy. Although the placental tissue was not preserved long enough to allow for pathological analysis again, the clinical progression strongly supported a definitive diagnosis of IC. The patient underwent 6 courses of multi-agent chemotherapy (etoposide, methotrexate, actinomycin D, cyclophosphamide, and vincristine), then her β-hCG level successfully decreased to within the normal range. Throughout this period, the infant’s β-hCG levels remained negative and there was no significant change in IPSVS. To date, both the patient and her infant are in good health.

## 3. Discussion

To date, few cases of IC coexisting with FMH have been reported in the literature. In some cases, it has been reported as appearing in combination with other conditions such as maternal disseminated intravascular coagulopathy, or as having its onset in twin pregnancy.^[4,5]^ IPSVS, a rare malformation of circulatory system, was detected incidentally in the last ultrasound examination, making the diagnosis more difficult. To the best of our knowledge, this is the first case report of IC co-occurring with FMH in which the fetus also had IPSVS. Whether the simultaneous occurrence of IC and IPSVS is related deserves further investigation. However, when reviewed in the context of other reported cases, the patient’s COVID-19 infection during early pregnancy appears to have been an independent risk factor for the co-occurrence of multiple abnormalities.^[6]^

During the development of IC, the malignant trophoblastic tissue invades the intervillous space, including the uterine wall and maternal vascular space. This can cause extensive release of fetal blood into the maternal circulation, leading to FMH. Decreased fetal movement, sinusoidal fetal heart rate tracing and increased MCA-PSV are the most important manifestations of FMH prenatally.^[7]^ Measurement of MCA-PSV by Doppler ultrasonography has become the standard for noninvasive diagnosis of fetal anemia, and PSV value is inversely proportional to fetal hemoglobin level within a certain range.^[8]^ However, if the hemoglobin concentration is reduced substantially to 10 to 30 g/L, this relationship is disrupted, and PSV will not increase proportionally.^[8,9]^ Thus, if MCA-PSV exceeds 1.55 multiples of the median, the potential for severe anemia should be considered and that FMH should be included in cause analysis.

IPSVS, with limited known association to fetal anemia, was the biggest confounding factor in etiological analysis of fetal anemia in this case. Theoretically, the shunt channel allowed oxygen-rich blood in the portal vein to bypass the liver and drain directly into the systemic circulation, resulting in nutrient deficiency in the liver. Because the liver is the main site of fetal hematopoiesis, its underdevelopment has the potential to cause fetal anemia. By contrast, increased oxygen-rich blood in the systemic circulation facilitated by the presence of the shunt channel may have alleviated fetal hypoxia to some extent. As the degree of fetal anemia was underestimated to some extent, IPSVS was considered as the culprit for fetal anemia initially.

According to the published literature, 50% of IC diagnoses are incidental, meaning that half of IC cases occur in women with uneventful pregnancies.^[2]^ Furthermore, pathological examination of the placenta has limitations, thus more consideration is needed regarding how to avoid missed diagnosis of IC.^[2]^ Ultrasound combined with determination of β-hCG level is the main method for the diagnosis of GTD. The characteristic ultrasonic manifestation of GTD includes increased echogenicity and the presence of small cystic changes within the placenta. However, due to the small size of the lesion, it’s extremely challenging to directly identify the abnormality, especially in late pregnancy cases. Fortunately, the manifestations of its complications are easier to be detected and provide important diagnostic clues for the occurrence of IC, such as FMH. Once IC is suspected, β-hCG level should be measured, and more targeted examinations can be conducted to confirm diagnosis. In this case, although FMH was diagnosed shortly after delivery, a lack of experience led to the oversight of IC as a potential cause, resulting in the delayed diagnosis of IC. This is a valuable lesson to be learned from this case.

## 4. Conclusion

This case enriches the available data regarding IC coexisting with FMH and is the first report of a co-occurrence of fetal IPSVS. Although rare, unexplained FMH warrants consideration of IC as a potential underlying cause. For patients with FMH, thorough placental pathological examination and postpartum HCG monitoring are essential to prevent potential misdiagnosis. Enhancing the understanding of the prenatal and postnatal manifestations of IC and strengthening multidisciplinary cooperation are of great significance for its early diagnosis.

## Acknowledgments

We thank Jane Bryant, PhD, from Liwen Bianji (Edanz; www.liwenbianji.cn) for editing the English text of a draft of this manuscript.

## Author contributions

**Conceptualization:** Yi Sun, Pei Zhou.

**Data curation:** Xingxing Wang.

**Investigation:** Yongpan Tan, Tao Liu.

**Writing – original draft:** Yi Sun, Pei Zhou.

**Writing – review & editing:** Lufang Wang.
